# A Comparative Study on the Antidiabetic Activity, Cytotoxicity and Lipid Profile of *Trichilia emetica* Oils

**DOI:** 10.3390/plants13162234

**Published:** 2024-08-12

**Authors:** Mohammed Aldholmi, Ebtihal Althomali, Fatema Aljishi, Rizwan Ahmad, Aljawharah Alqathama, Deema Alaswad

**Affiliations:** 1 Department of Natural Products, College of Clinical Pharmacy, Imam Abdulrahman Bin Faisal University, Dammam 31441, Saudi Arabia; 2 Department of Pharmaceutical Sciences, Faculty of Pharmacy, Umm Al-Qura University, Makkah 21955, Saudi Arabia

**Keywords:** palmitic acid, oleic acid, GC-MS, mafura, natal mahogany

## Abstract

The *Trichilia emetica* plant is traditionally used for medicinal and food purposes. However, there are limited studies on the bioactivity and cytotoxicity of its seed butter and aril oil. This study aimed to assess the antidiabetic activity and cytotoxicity of seed butter and aril oil, obtained via two different extraction methods, and compare their lipid profiles. The plant samples were collected from the Faifa mountains and extracted using a Soxhlet apparatus for hot extraction and a magnetic stirrer for cold maceration. The antidiabetic activity and cytotoxicity were evaluated using the α-amylase and MTT assays, respectively. The fatty acids were quantified utilizing gas chromatography-mass spectrometry. This study proves the impact of the extraction method on the yield, cytotoxicity, antidiabetic activity and lipid profile. The highest cytotoxicity was observed with the seed butter obtained via Soxhlet extraction. The α-amylase inhibition was observed at the highest levels with the seed butter and aril oil obtained via cold maceration. The palmitic acid (PA) and oleic acid (OA) were detected at their maximal concentrations in the seed butter obtained via Soxhlet extraction and aril oil obtained via cold maceration, respectively. This study represents an essential basis for understanding the importance of *T. emetica* as a valuable tree for food, cosmetic and medicinal purposes. Further experiments can lead to the development of green extraction techniques and isolation of the cytotoxic and antidiabetic molecules that can be developed into new pharmaceutical products or serve as lead molecules for new drugs.

## 1. Introduction

*Trichilia emetica Vahl* (*T. emetica*) is a species within the *Trichilia* genus of the Meliaceae family [1]. It is indigenous to certain African countries, Saudi Arabia, and Yemen [2]. It is the only species of the *Trichilia* genus that has been described in Saudi Arabia [3,4]. This species has been identified in different areas of the Kingdom of Saudia Arabia, such as Jabal Fayfa (the Faifa mountains), Wadi Dahwan, Jabal Abadil and Wadi Lejib [4]. *T. emetica* is commonly known as the Mafura tree or Natal Mahogany in Africa, while in Arabic it is known as Wasaf, Umshara and Um Hagi [5]. The tree is characterized as an evergreen with a bark that can be either smooth or rough, appearing in shades of gray or brown [1,5]. The fruit is round and has a furry appearance, featuring a red or brown color [5]. It comprises capsules that split into three or four parts, revealing 3–6 shiny black seeds [5]. Each seed is covered by an orange-to-red aril [5]. *T. emetica* is easily identified and distinguished from *T. dregeana*, commonly known as forest mahogany, by observing some basic features of the leaves and fruits [6]. The leaves of the tree contain approximately 15 pairs of lateral veins with a rounded and notched apex, whereas only 8–12 are evident in *T. dregeana* with acute to acuminate tips, and the leaves of the former are less glossy and not as dark in color as those of *T. dregeana*. The fruit capsules of *T. emetica* are pale green compared to the pinkish-green fruit capsules of *T. dregeana*. *T. emetica* fruits yield two distinct types of oil: “mafura oil”, extracted from the fleshy seed envelope or aril and suitable for human consumption, and “mafura butter”, obtained from the seed kernel, possessing a bitter and astringent taste that makes it inedible [7].

The name *T. emetica* is derived from its emetic properties, evident in the traditional use of root and bark infusions and mafura butter [1,7]. Beyond its emetic properties, *T. emetica* holds various roles in traditional medicine. For instance, the root decoction is used as a diuretic, a labor inducer and for treatment of jaundice and infertility [1,8]. The leaves are commonly used for wounds, burns and bruises [5,9]. In addition, both *T. emetica* bark and leaf infusions are utilized to alleviate backaches [5]. The seeds of *T. emetica* hold significant value in cosmetic practices, yielding high-quality butter and oils [5]. These are essential in manufacturing various products like soaps, hair conditioners and candles [5,7]. Moreover, the seed oils are used for promoting wound healing and for treating infectious diseases such as leprosy [1,5]. Internally, the seed oil is ingested to treat rheumatism [9]. The aril oil is used in food preparation both as a cooking oil and as a flavoring agent [5].

The phytochemical profile of *Trichilia* is composed mainly of terpenoids, including triterpenes, sesquiterpenes, limonoids, steroids and polyphenols [10]. Different limonoids have been isolated from the barks of *T. emetica*, including nymania 1, drageana 4, trichilin A, trichilin C, trichilin D, aphanastatin, rohituka 3, Tr-B, seco-A protolimonoid and 7-acetyltrichilin A [11]. It has also been suggested that the seeds of *T. emetica* may contain a limited amount of limonoids [8]. Another phytochemical class that has been detected in both the bark and seeds of *T. emetica* is that of flavonoids [8,12]. Naringenin, taxifolin, 4′-O-β-D-glucopyranoside, elephantorrhizol, catechin 3-O-β-D-glucopyranoside and eriodictyol 3-O-β-D-glucopyranoside are among the flavonoids isolated from the seeds of *T. emetica* [13,14]. Moreover, several studies have explored the fatty acid composition in both seed and aril parts [7,15,16]. The majority of studies consistently observed that the saturated fatty acid (SFA) palmitic acid (PA) and the monounsaturated fatty acid (MUFA) oleic acid (OA) are the major components of both seed and aril oils, representing over 88% of the fatty acid content [15,16]. Additionally, linoleic acid and stearic acids have been reported in both parts [7,15,16]. However, cis-vaccenic acid is exclusively found in seed butter [15]. Arachidic acid and linolenic acid are detected in low amounts in aril oil [16].

The extensive traditional uses of *T. emetica* encouraged scientists to investigate a diverse range of the biological activities of different parts of the plant [5]. Numerous pharmacological studies indicated that the extracts from the root, leaves and stem bark of *T. emetica* showed antibacterial activity against different pathogens [12,17,18,19]. Similarly, previous studies demonstrated that the extracts derived from the fruit, leaf, stem bark and seed of *T. emetica* displayed antifungal activity [18,19,20,21]. Several studies have also reported the antioxidant properties of extracts obtained from the stem bark, root bark, and seed of the *T. emetica* plant [12,20]. Its polyphenolic compounds have been shown to be responsible for the high antioxidant activity of *T. emetica* root in rat liver microsomes [22]. The aqueous extract of *T. emetica* leaves exhibited analgesic activity with a 75% reduction in pain [23]. Additionally, the aqueous extract of *T. emetica* leaves and root bark demonstrated an anti-inflammatory effect with edema reduction [23]. The leaf extract has also been shown to have antidiabetic activity via the inhibition of the α-amylase enzyme [24]. The α-amylase enzyme is a glycoside hydrolase that aids digestion by breaking down carbohydrates via hydrolyzing the glycosidic bonds of polysaccharide molecules to produce simpler carbohydrates, leading to an increase in postprandial glucose levels [25]. Therefore, this enzyme is a key therapeutic element that has been exploited for the prevention and management of diabetes through decreasing diet-dependent blood glucose levels [26]. In terms of cytotoxic activity, Kurubasch aldehyde, a sesquiterpenoid isolated from the roots of *T. emetica*, has been found to decrease the proliferation of breast cancer cells (MCF-7) and inhibit the proliferation of murine sarcoma S18 cancer cells [27]. The seeds are toxic to cattle, and studies have reported that the other parts of the plant are very toxic [5]. However, it’s not clear if the toxic compounds are present in the aril or seed part due to a lack of comparative cytotoxic studies of the seed and aril extracts.

As discussed above, both various bioactivities and cytotoxic activity have been documented for the bark, leaves, roots, and seeds of *T. emetica.* Nevertheless, mafura butter from its seeds has only been investigated for antifungal and antioxidant activity [20]. Moreover, the mafura oil from its aril part has not been evaluated for its bioactivity nor its cytotoxicity. Therefore, this study aimed to assess the antidiabetic activity and cytotoxicity of both mafura butter and mafura oil and compare their lipid profiles using PA and OA as chemical markers. Soxhlet extraction and cold maceration with hexane are commonly employed for the extraction of *T. emetica* oils [7,15,16,28]. Hence, these methods were utilized for extraction in this study, but utilizing a shorter time (4 h) for hot extraction. The extraction time was estimated by monitoring the color of the solvent in the siphon and comparing the total yield to previous studies. Previous studies have reported conflicting results about the dominant fatty acid in *T. emetica*, with some reporting PA as the dominant fatty acid and others suggesting OA as the major one [7,15,16,29]. This inconsistency motivated the authors of this paper to conduct a head-to-head comparison of seed butter and aril oil via the quantification of PA and OA.

## 2. Results and Discussion

### 2.1. Extraction Yield from Seed and Aril

The oils obtained from the seed via hot (HS) and cold (CS) extraction were solid at room temperature, and the yield was 41% for the former method of extraction and 34% for the latter. The oils obtained from the aril were liquid at room temperature, and the yields were 28% and 22% for the hot (HA) and cold (CA) extractions, respectively. In this study, it was proven that the Soxhlet extraction method is the optimal method for the obtainment of the highest quantities of both seed and aril oils. The seed provided the highest yield of oil compared to the aril part. This is comparable to previous studies on *T. emetica* oils, which described the seed oil as solid oil (butter), with an average extraction yield ranging between 42% to 50% [28,30], and the aril oil as liquid oil, with an average extraction yield ranging from 28% to 30% [7,30]. However, there was no clear justification for the variation in the yield, but it is presumed that the preparation and extraction methods had a considerable effect on the yield and quality of the extracted oils. In this study, the shortest possible extraction time (4 h) was used in the Soxhlet extraction, to save time and minimize the effect of heating on oil components, while more extended extraction periods were used in previous studies. The more prolonged extraction periods might increase the oil yield, but do so at the cost of its quality. The country of origin and its climatic and ecological conditions could be another important factor for the variations in oil quantity and quality. It has been reported that the seeds from *T. emetica* growing in East Africa provided higher yield of oil than those coming from a West African tree [9].

Interestingly, the average extraction yield of *T. emetica* oil in the current and previous studies is higher than that of some edible oils, such as soybean and linseed. The content of soybean oil obtained via Soxhlet extraction with hexane for 150 min was approximately 21.4% [31]. However, the heating temperature used in the extraction process was not reported. The same study reported a 14.9% yield when extracting soybean via maceration with hexane for 150 min. The extraction of linseed via the Soxhlet apparatus with hexane for 14 h at 60–80 °C provided a yield of 36.12% [32]. However, the extracted oils were not treated with a drying agent, which might lead to overestimation of yield. Additionally, it is necessary to remove any precipitates in the oil before calculating the total yield. In our study, the resulting oils were treated with magnesium sulfate and centrifuged at 4000 rpm to obtain the accurate yields of seed butter and aril oil. The yields in grams and extraction yield (%) results are summarized in Table 1.

### 2.2. Antidiabetic Activity T. emetica Oils

**Figure 1:**
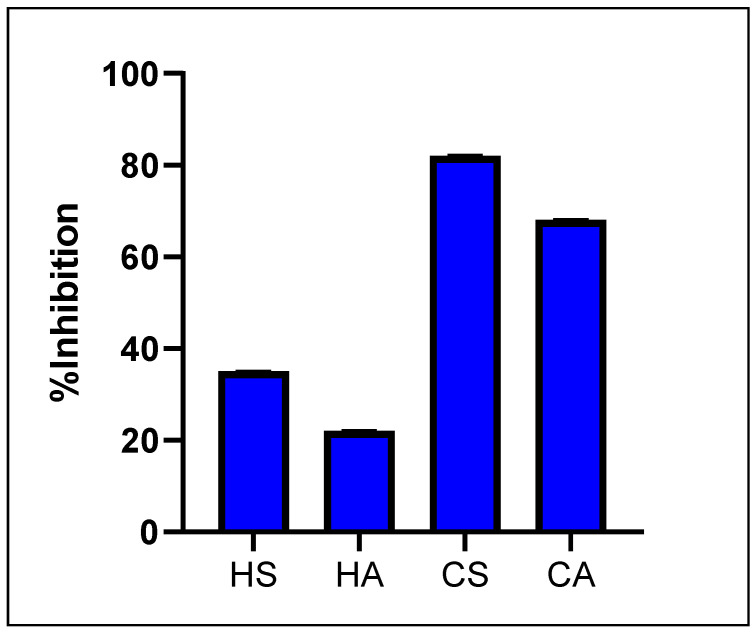
α-Amylase inhibitory activity of the tested extracts (% of enzyme inhibition ± SD μg/mL), 500 μg/mL.

The initial screening of oil extracts in a phosphate buffer (500 μg/mL) for α-amylase activity revealed inhibition of less than 50% for both butter and oil obtained via the hot extraction method of the *T. emetica* seed (35 ± 0.15%) and the aril (22 ± 0.11%), while those obtained via cold maceration of the *T. emetica* seed and the aril presented 82 ± 0.17% and 68 ± 0.19% respectively, as shown in Figure 1. This notable difference in bioactivity between the hot and cold extracts might be a result of a degradation of the heat labile α-amylase inhibiting phytochemicals during the process of hot extraction at 70 °C for 4 h, which is avoided in the cold maceration with the magnetic stirrer.

**Figure 2:**
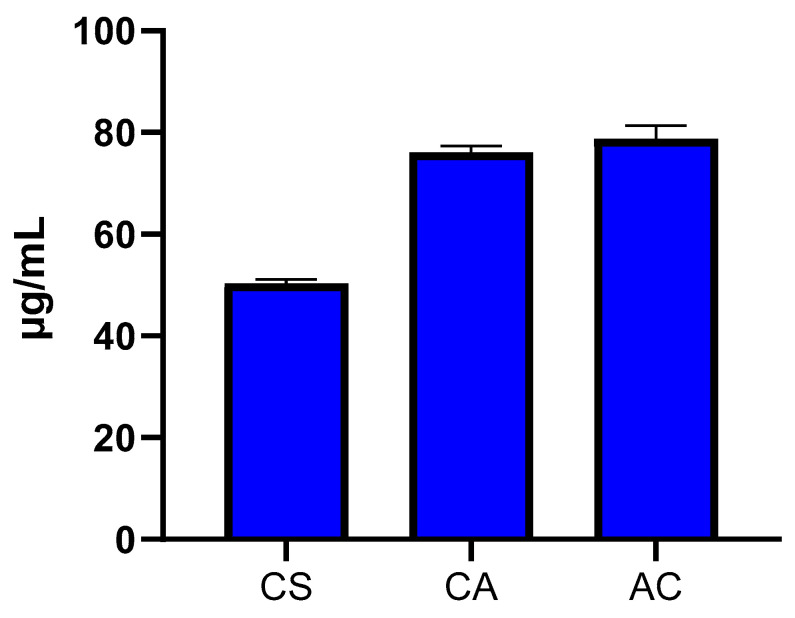
α-Amylase inhibitory activity of the selected extracts (IC_50_ ± SD μg/mL).

The butter and oil samples with higher than 50% inhibition (CS and CA) were further investigated at seven different concentrations (5–1000 μg/mL) for the determination of the IC_50_ values in comparison to acarbose (AC), a well-known α-amylase inhibitor. Interestingly, the mafura butter of the seed appeared to be a more potent inhibitor of α-amylase enzyme (50.26 ± 0.90 μg/mL) than acarbose (78.82 ± 2.52 μg/mL), while the aril oil showed a comparable level of inhibition (76.10 ± 1.31 μg/mL) to acarbose (Figure 2). A previous study has reported that the leaf extract of *T. emetica* presented antidiabetic activity via the inhibition of the α-amylase enzyme [24]. However, the other parts of the plant, including its butter and oil, have not been investigated for antidiabetic activity, particularly the α-amylase inhibition activity. Botanical extracts and oils from the *Meliaceae* family have been reported to exhibit potent α-amylase inhibition activity [33,34,35]. For example, *Carapa procera*, typically called African crabwood, is a popular plant from the *Meliaceae* family with α-amylase and α-glucosidase inhibition activity [35]. Similarly, the seed oil of *Azadirachita indica*, typically known as neem oil, exhibited inhibitory effects against several enzymes, including α-amylase, α-glucosidase, tyrosinase and lipase [33]. Additionally, the aqueous extract of neem displayed α-amylase inhibitory activity with an IC_50_ value of 9.15 mg /mL [34]. However, the reported bioactivity of this oil is significantly lower than that of *T. emetica* seed butter and aril oil as observed in this study. Therapeutic inhibitors of the α-amylase enzyme such as acarbose have been clinically used for the management of hyperglycemia, but they cause patients to suffer from several side effects, such as flatulence, diarrhea and abdominal discomfort [26]. Hence, the discovery and development of new α-amylase inhibitors with minimal side effects can be advantageous for the management of hyperglycemia in Type 2 diabetes patients. The butter and oil of *T. emetica* can be further investigated as a potential treatment or a source of drugs for the treatment of hyperglycemia.

### 2.3. Cytotoxicity of T. emetica Oils

**Figure 3:**
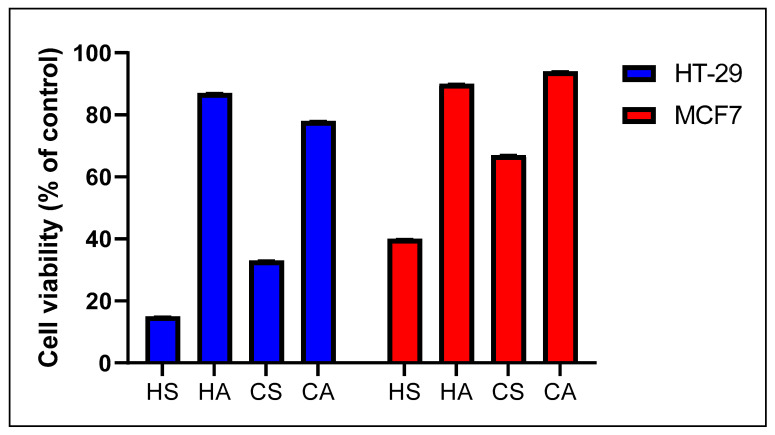
Cytotoxicity of the tested extracts against two cell lines (MTT 48 h, % of cell viability ± SD), 100 μg/mL concentration.

The lowest cytotoxicity was noticed with the aril extracts (HA and CA) obtained via both hot and cold maceration methods, showing cell viability between 78 ± 0.15% and 94 ± 0.12% (Figure 3). The seed extract obtained via cold maceration (CS) presented moderate cytotoxicity with 33 ± 0.14% cell viability in the colorectal adenocarcinoma cells (HT-29) and 67 ± 0.21% in the breast cancer cells (MCF-7). The highest cytotoxicity was observed for the seed extract obtained via hot extraction against HT-29 and MCF-7 cell lines with 15 ± 0.05% and 40 ± 0.05% cell viability, respectively. Therefore, this extract was further investigated for the determination of IC_50_ and selectivity in HT-29 and MCF7, as well as in fetal lung fibroblast cells (MRC-5) and liver cancer cells (HepG2), across a range of concentrations from 1 to 500 μg/mL, in comparison to the oxaliplatin and olaparib positive controls.

**Figure 4:**
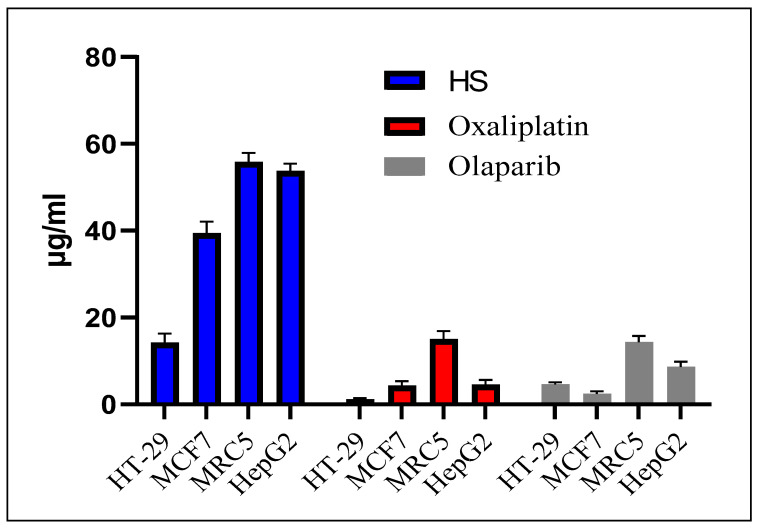
Cytotoxicity and selectivity of the selected extract (MTT 48 h, IC50 ± SD μg/mL) compared to oxaliplatin and olaparib.

The highest cytotoxicity and selectivity of seed hot extract was observed against HT-29 with an IC_50_ ± SD of 14.24 ± 2.07 μg/mL, compared to 39.41 ± 2.70, 55.82 ± 2.05 and 53.75 ± 1.66 μg/mL in MCF-7, MRC-5 and HepG2, respectively (Figure 4). However, it is worth noting that the IC_50_ of the extract against the normal cell line MRC-5 (55.82 ± 2.05) is four times that against the colorectal cell line, suggesting higher selectivity of the extract against the cancer cell lines than the normal cells. Even though the cytotoxic activity of *T. emetica* seed hot extract is about 12 times the cytotoxic dose of oxaliplatin (1.18 ± 0.25) and three times that of olaparib (4.62 ± 0.49), it can be considered a highly cytotoxic extract, considering that this is a crude extract containing several inactive components. This cytotoxicity is significantly higher than that reported for the seed extract of *A. indica*, from the *Meliaceae* family [36]. The cytotoxicity of *A. indica* seed methanolic extract has been evaluated against four cancer cell lines (MCF-7, HepG2, A549 and HT-29) and one normal cell line (MDBK). The seed extract presented moderate cytotoxicity with an IC_50_ of 270.58, >650, 370.69, 343.28 and >650 μg/mL against MCF-7, HepG2, A549, HT-29 and MDBK, respectively [36]. However, the leaf extract exhibited potent cytotoxicity with an IC_50_ of 34.11 against MCF7 and 38.44 μg/mL against HT29 cancer cell lines. Various cytotoxic compounds have been isolated from *A. indica*, such as azadirachtin, nimbolide, azadirone, azadiramide A, 1-O-deacetylohchinolide B and 15-O-deacetylnimbolindin B [37]. Therefore, the preliminary results on the cytotoxic activity of *T. emetica* seed extract could motivate researchers to isolate and elucidate the structure of the bioactive compounds, which could lead to the discovery of novel chemical entities.

Previous studies have reported high cytotoxic activity in different parts of *T. emetica* [5], but cytotoxic studies of the butter and oil are lacking. The root has been reported to contain a sesquiterpenoid phytochemical named the Kurubasch aldehyde [27]. This compound has been found to decrease the proliferation of breast cancer cells (MCF7) and inhibit the proliferation of murine sarcoma S18 cancer cells. Previous reports have revealed toxicity in animals due to their feeding on *T. emetica* seeds, particularly in cattle [5]. However, it was not evident if the toxic compounds are present in the aril or kernel part due to a lack of comparative cytotoxic studies of the seed and aril extracts. This study answered this question by comparing the cytotoxicity of the butter and oil obtained from the seed and aril, respectively. The cytotoxic activity was significantly higher in the seed butter than in the aril oil. Moreover, the hot extraction method resulted in a higher cytotoxic activity compared to the cold maceration. This can be attributed to the higher efficiency of Soxhlet extraction compared to the cold maceration method. In Soxhlet extraction, the solvent is continuously vaporized, resulting in multiple cycles of extraction with a fresh solvent in each cycle which displaces the mass transfer equilibrium and enhances the solubility of extracted compounds [38]. Additionally, this extraction method uses heat to vaporize the solvent, which can reach the extracted samples and help in extraction. The cold maceration method has a higher possibility of mass transfer equilibrium due to the direct continuous contact between the solvent and extracted materials. However, this method could be advantageous for the extraction of heat-labile bioactive compounds.

### 2.4. Fatty Acid Derivatization and GC-MS Analysis

The optimal fatty acid derivatization was found by adding 20 mg each of PA and OA to a 4 mL methanolic solution containing 12% boron trichloride (BCl3) and 10% of 2,2-diemtoxypropane and then heating at 70 °C for 10 min, followed by the addition of 2 mL of water and 2 mL of hexane to the reaction tube after cooling. The separation of the derivatized PA and OA was accomplished in less than 10 min (Supplementary Figure S1). The GCMS method exhibited an accuracy ± SD of 97.63 ± 11.37% for palmitic acid (PA) and 101.5 ± 10.5% for oleic acid (OA) in the linearity range of 0.5–100 ppm (0.5, 1, 5, 10, 50, 100).

### 2.5. Content of OA and PA in T. emetica Oils

**Figure 5:**
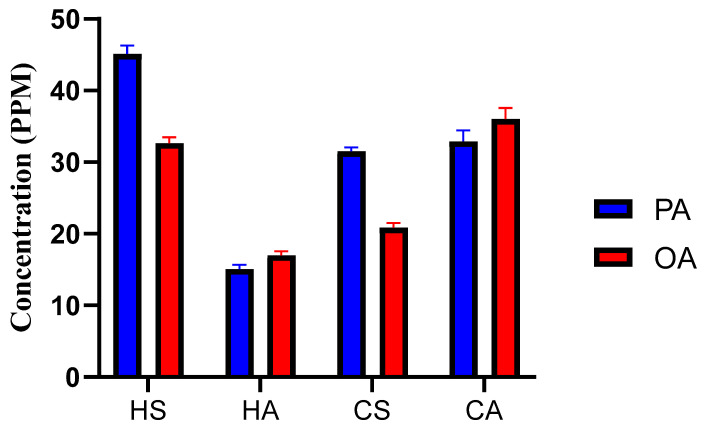
Concentrations of palmitic acid (PA) and oleic acid (OA) in hot (HS and HA) and cold (CS and CA) extracts.

The seed butter contained higher concentrations of PA than OA (approximately 1.5:1) while the aril oil contained slightly higher concentrations of OA than PA (approximately 1.1:1), when using both hot and cold maceration methods (Figure 5). However, the concentrations of both PA and OA in the seed butter obtained via the cold maceration method were considerably lower than those in the same sample obtained via hot extraction. In contrast, the aril oil obtained via cold maceration contained higher quantities of the two fatty acids when compared to the oil obtained via hot extraction. This can be attributed to the effect of heating on the fleshy part, which is a delicate, heat-sensitive material compared to the strong seeds, which require harsh conditions to release the fatty acids. Previous studies have reported conflicting results about the dominant fatty acid in *T. emetica*, with some reporting PA as the dominant fatty acid and others suggesting OA as the major one [7,15,16,29]. However, this study reports that the PA is the dominant fatty acid in the seed butter and the OA is the dominant one in the aril oil, regardless of the employed extraction method. The presence of high quantities of the saturated fatty acid (PA) in the seed butter is consistent with its solid state, in opposition to the liquid aril oil that contains high amounts of the monounsaturated fatty acid (OA). Additionally, the dominance of OA in the aril oil makes it preferable to the seed butter for the internal consumption. The substitution of saturated fat rich oils with oleic acid rich oil has been described to reduce the risks of cardiovascular diseases by decreasing the circulating concentration of the low-density lipoprotein (LDL) and increasing the beneficial high-density lipoprotein (HDL) cholesterol concentration in the blood [39]. Therefore, the aril oil can be developed as a commercial, healthy, edible oil with similar properties to the other oleic acid-rich oils, such as olive oil.

### 2.6. Descriptive Statistics

The statistical analysis revealed (*n =* 4) a sum with a mean (±SD) of 125.9% and 31.48 (±8.20) for the extract yield, 213.0 and 53.25 (±34.75) for the cell viability in HT-29, 291.0 and 72.75 (±24.86) for the cell viability in MCF-7, 207.0 and 51.75 (±27.95) for α-amylase inhibition, 124.6 and 31.15 (±12.33) for PA concentration and 106.5 and 26.62 (±9.14) for OA concentration. The ranges observed were 22.2–41.5 (extract yield), 15.0–87.0 (HT-29), 40.0–94.0 (MCF-7), 22.0–82.0 (α-amylase), 15.1–45.1 (PA) and 17.0–36.0 (OA).

### 2.7. Pearson’s Analysis

**Figure 6:**
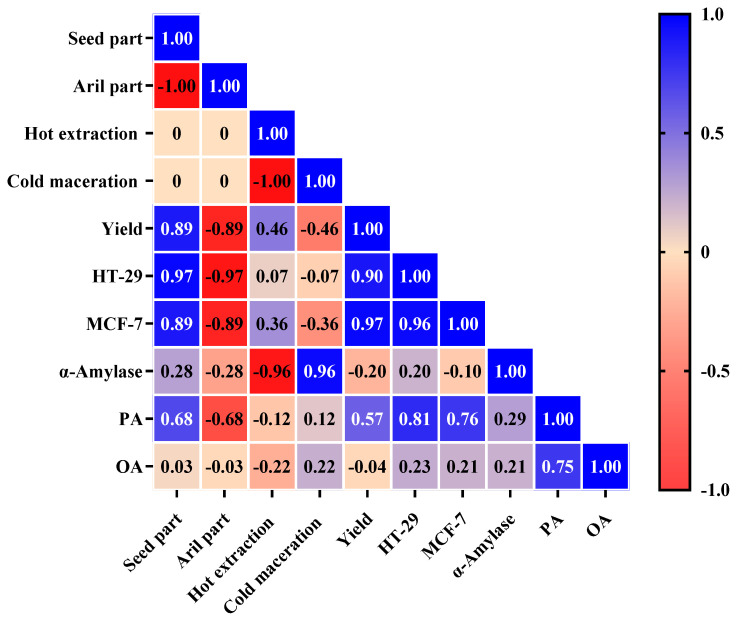
An illustration of correlation coefficients for the dependent variables.

The Pearson coefficient analysis was employed to measure the strength of the linear correlation between the variables, with coefficients ranging from −1 for the strongest negative correlation to +1 for the strongest positive correlation (Figure 6). The variables with a coefficient of 0 present no linear correlation. Coefficients with an absolute value of ≥0.70 were considered as showing strong correlations; those with a value of 0.40–0.69, moderate correlations; and those with a value of <0.40, weak correlations [40]. The seed part presented a strong positive correlation with the yield and with cytotoxic activity against HT-29 and MCF-7 cancer cell lines. This part also presented a moderate positive correlation with PA and a negligible correlation with OA. On the contrary, the aril part showed a strong negative correlation with the yield and with cytotoxic activity against HT-29 and MCF-7 cancer cell lines, a moderate negative correlation with PA and a negligible correlation with OA. Both the seed and aril parts demonstrated a weak correlation with α-amylase enzyme inhibition activity. This confirms that the extracted part has a significant impact on its cytotoxic activity and indicates that the cytotoxic compounds are present in abundant amounts in the seed part. The Pearson analysis also confirms the higher yield of seed butter compared to aril oil, which is consistent with previous studies [7,28,30]. The hot extraction method presented a strong negative correlation with α-amylase enzyme inhibition activity whereas cold maceration showed a strong positive correlation with α-amylase. Both hot extraction and cold maceration presented a weak to moderate correlation with the other variables. The strong correlation between the extraction method and α-amylase indicates the influence of the heating during hot extraction on the α-amylase enzyme inhibitors, which appear to be present in both the seed and aril parts. Therefore, the employed extraction method can differ depending on the required biological activity. A strong positive correlation was observed between the yield and cytotoxic activity against both cancer cell lines. There was also a strong positive correlation between the PA content and cytotoxic activity. Although PA serves as an energy source and a building-component in human cells, it has been shown to cause apoptotic cell death and mitochondrial damage [41]. It has also been reported to decrease cell viability and ATP production in human cancer cells without an impact on human normal cells [41,42]. Interestingly, monounsaturated fatty acids (MUFA) such as OA have been reported to protect cells against a PA-induced decrease in cell viability [43]. PA has also been tested in mice and found to display an in vivo antitumor activity [42]. However, the cytotoxicity reported in this study might be associated with several bioactive components of the extract.

## 3. Materials and Methods

### 3.1. Materials and Plant Preparation

HPLC-grade n-hexane, BCl3-methanol (12% *w*/*w*), 2,2-dimethoxypropane and fatty acid standards (OA and PA) were purchased from Sigma-Aldrich (Darmstadt, Germany). The mature, newly opened fruits of *T. emetica* were collected between August and September 2022 in the Faifa mountains of Jizan, Saudi Arabia, and identified by the natural products department of Imam Abdulrahman Bin Faisal University, Dammam, Saudia Arabia, where a voucher specimen was deposited. After identification, the whole seeds were dried in the sun before the flesh (aril) was manually separated from the inner part (seed). The aril and seed were ground using an electric blender immediately before Soxhlet extraction and cold maceration.

### 3.2. Extraction and Determination of Oil Yield

Two extraction methods (hot and cold) were used to compare the effect of temperature on the lipid profile and biological activity of the oils obtained from the aril and the seed of *T. emetica*. The hot extraction was performed using 220 mL of hexane to extract 24 g of aril or seed in a Soxhlet apparatus for 4 h. The cold maceration was conducted via magnetic stirring of 24 g of aril or seed in 220 mL of hexane at room temperature for 24 h. The solvent was removed using a rotary evaporator, and the resulting oils were treated with magnesium sulfate and centrifuged at 4000 rpm to remove precipitates. The oils were transferred through filter paper to previously weighed empty tubes before weighing the tubes again to calculate the yield. The percentage yield of oils was determined using the following equation:

Oil% = Weight of the oil extract/Weight of the extracted sample × 100

### 3.3. Antidiabetic Activity

The extracts’ antidiabetic activity was evaluated by measuring the inhibitory effect of extracts on the α-amylase enzyme. All extracts were initially tested at 500 μg/mL, and those that demonstrated an inhibitory effect larger than 50% were further investigated at a range of concentrations (1000, 500, 250, 100, 50, 25, and 5 μg/mL), with acarbose as a positive control. The inhibitory activity of ɑ-amylase was ascertained as previously described [44]. An aliquot of 1 mg of α-amylase from *Aspergillus oryzae* in a phosphate buffer was prepared, and 20 μL was added to each well in a 96-well plate, along with 20 μL of the extract samples that were diluted in a phosphate buffer. After mixing, the plate was incubated for 10 min at 37 °C, then 30 μL of starch (0.05% in deionized water) was added to each reaction well and incubated for a further 8 min at 37 °C. The reaction was then halted by adding 20 μL of hydrochloric acid (1 M) and 100 μL of iodine reagent (0.25 mM) to each well. Control wells were set up by replacing the enzyme with buffer and adding acarbose to create a positive control blank. Negative control wells contained the reaction mixture with 20 μL of phosphate buffer containing the vehicle solvent (DMSO 0.4%). Absorbance was measured using a multi-plate reader (BIORAD, PR 4100, Hercules, CA, USA) at 550 nm for each well, calculating the percentage of inhibition using the equation

% inhibition = (A − C/B − C) × 100

where A = the absorbance of the reaction mixture in the presence of the extract, B = the absorbance of the mixture without the enzyme and C = the absorbance of the reaction mixture in the absence of any extract.

### 3.4. Cell Culture and Cytotoxicity

Three cancer cell lines, MCF-7 (human breast adenocarcinoma, ATCC-HTB22), HT-29 (human colon adenocarcinoma, ATCC-HTB-38™), HepG2 (hepatocellular carcinoma, ATCC HB-8065), and one normal fibroblast cell line, MRC-5 (normal human fetal lung fibroblast, ATCC-CCL171), were utilized in this study. Three of the cell lines (MCF-7, HT-29 and MRC-5) were maintained in Roswell Park Memorial Institute Medium (RPMI-1640, Gibco, Life Technologies, Carlsbad, CA, USA), while the HepG2 cell line was cultured in Dulbecco’s Modified Eagle Medium (DMEM, Gibco, Life Technologies, Carlsbad, CA, USA). All of the cell lines were maintained at 37 °C in 5% CO_2_, and 100% relative humidity. All media were supplemented with 10% heat-inactivated fetal bovine serum (FBS, Gibco) and 1% penicillin-streptomycin antibiotic, consisting of 10,000 units of penicillin and 10,000 µg of streptomycin (Gibco) per mL.

The cytotoxicity of the extracts was evaluated via viability MTT assay, as previously reported [45]. The MCF-7, HT-29, HepG2 and MRC-5 cell lines were tested to determine the cytotoxicity and selectivity of the extracts. The MCF7 and HT-29 cells were cultured separately in 96-well plates (3 × 10^3^/well) and incubated at 37 °C overnight as a screening stage for the cytotoxic activities of the extracts. The first set of experiments tested the extracts at a 100 μg/mL concentration (DMSO 0.4%; *n* = 3). Plates were left at 37 °C in 5% CO_2_ for 48 h, after which MTT was added to each well, and the plates incubated for a further 3 h. The supernatant was removed, and the MTT crystals were solubilized by adding DMSO to each well. Absorbance was read using a multi-plate reader (BIORAD, PR 4100, Hercules, CA, USA). The optical density of the purple formazan A550 was proportional to the number of viable cells, which was calculated as an inhibition percentage compared to control cells. Extracts with the highest percentage of inhibition were selected for the second set of experiments to determine cytotoxicity IC_50_ and selectivity, using the cancer cell lines as well as the fibroblast cell line. The extract concentrations tested were 500, 250, 100, 50, 10, and 1 μg/mL. Oxaliplatin and Olaparib were used as the positive controls while cells treated with 0.4% DMSO were used as the negative controls.

### 3.5. Samples Preparation and Derivatization

Fatty acids are not volatile, and require derivatization in order to improve volatility and enable their analysis via gas chromatography–mass spectrometry (GC–MS). Hence, fatty acid methyl esters were prepared for palmitic acid (PA) and oleic acid (OA), as previously described [46], with some modifications. Different amounts ranging from 10 to 40 mg of the fatty acids were added to 1–4 mL BCl3-methanol, 12% *w*/*w*, with and without the water scavenger 2,2-dimethoxypropane, to determine the optimal derivatization method. The mixtures were heated at different temperatures for different periods before allowing them to cool. After cooling, 1–2 mL of water and 1–2 mL of hexane were added to the mixtures and vortexed. The upper organic layer (containing hexane) was then transferred to a clean vial and centrifuged at 4000 rpm for 10 min before GC–MS analysis. The optimal derivatization conditions were used to prepare concentrations from 0.01 ppm to 100 ppm to create the calibration curve for GC–MS analysis. *T. emetica* oils were subjected to the same conditions to quantify PA and OA in the samples using GC–MS.

### 3.6. GC-MS Analysis

The GC–MS analysis was performed on a Shimadzu-2010 plus gas chromatography (Kyoto, Japan) equipped with a split/splitless injector and coupled with a QP2010 Ultra MS detector (Kyoto, Japan). The column used for the application was a nonpolar Rxi-5ms capillary column (30 m × 0.25 mm, 1.00 μm; Restek Corporation, Bellefonte, PA, USA). The oven was initially maintained at 50 °C for 1 min and then ramped to 280 °C at a rate of 70 °C/min (held for 5.71 min). Helium was used as a carrier gas (1.5 mL/min). The temperatures for the ion source and MS transfer lines were maintained at 250 and 280 °C, respectively. Mass spectra (50–600 *m*/*z*) were acquired starting from 5 min and ending after 10 min. Shimadzu GCMS Solution^®^ (V. 4.52) software (Kyoto, Japan) was used for data acquisition, processing and GC–MS control. Compound identification was achieved via mass spectral searching within the NIST11 Library database and comparison to the spectra of external PA and OA methyl esters. The target analytes were quantified using external standards calibration.

### 3.7. Statistical Analysis

SPSS V 22.0 and GraphPad Prism 8 were used to analyze the data and to create the graphs. The results are expressed as the mean ± standard deviation (SD) from at least three independent experiments.

## 4. Conclusions

This head-to-head comparative study proved the effects of the extraction methods on the resulting yields, cytotoxicity, α-amylase inhibition and fatty acid concentrations. The extraction method used significantly affected the yields of the seed butter and the aril oil, providing higher butter and oil yields when the hot extraction method was employed. Additionally, the yield was influenced by the extracted part, with the seed being the source with the highest extract yield. Cytotoxicity was mainly noticed with the seed butter, more significantly so with the seed butter obtained via hot extraction. Inhibition of α-amylase was observed at the highest levels with the seed butter and aril oil obtained via cold maceration, but more significantly so with the former extract. The PA and OA were detected at their maximal concentrations in the seed butter obtained via hot extraction and aril oil obtained via cold maceration, respectively. Hence, the seed butter samples obtained via the hot extraction and cold maceration methods are the best candidates in terms of the extract yield when cytotoxicity is not a concern for consumers, particularly for skin care products. For oral use, the aril oil obtained via cold maceration is the most suitable extract as it contains high quantities of the beneficial monounsaturated fatty acid in addition to the α-amylase inhibition activity, which might be desirable for the control of hyperglycemia. The aril oil obtained via hot extraction might not be recommendable for any use due to low yield and lack of biological activities. The conclusions of this study represent an essential basis for understanding the importance of *T. emetica* as a valuable tree growing wildly in Saudi Arabia that can be exploited for food, cosmetic and medicinal purposes. Additionally, this study assists in the discovery of the cytotoxic part of the plant and the methods that result in the recovery or omission of the cytotoxic molecules during extraction. Further studies could lead to the isolation of the cytotoxic and antidiabetic molecules that could be developed into new pharmaceutical products, serving as lead molecules for new drugs. While the extraction techniques employed in this study exhibited high efficiency, they are not optimal in terms of solvent usage, time consumption and environmental sustainability. Therefore, green extraction techniques such as supercritical fluid extraction, ultrasound-assisted extraction and subcritical water extraction should be considered in order to develop a fast, ecofriendly and efficient extraction method.

## Figures and Tables

**Table 1:** Extraction yields of seed and aril oils obtained via hot (HS and HA) and cold (CS and CA) extraction methods.

Sample	Plant Material (g)	Oil Extracts (g)	Yield (Oil per g of Plant Material)	Yield (%)
HS	24	9.9	0.4125	41
HA	24	6.8	0.2833	28
CS	24	8.2	0.3417	34
CA	24	5.3	0.2208	22

## Data Availability

All data generated or analyzed during this study are included in this published article.

## Supplementary Materials

The following supporting information can be downloaded at: https://www.mdpi.com/article/10.3390/plants13162234/s1: Figure S1: GC-MS chromatogram of the derivatized PA and OA.
