# Supplementary figures and images for: A Comparative Study on the Antidiabetic Activity, Cytotoxicity and Lipid Profile of Trichilia emetica Oils
Source: Plants (Basel). 2024 Aug 12;13(16):2234. doi: 10.3390/plants13162234 (PMC11359310; doi:10.3390/plants13162234)

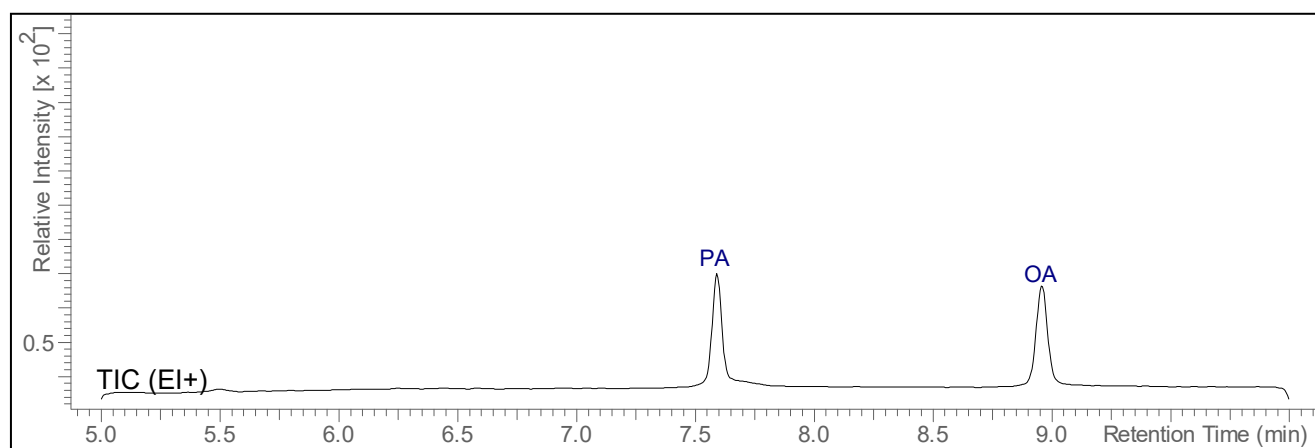

**Figure S1.** GC-MS chromatogram of the derivatized palmitic acid (PA) and oleic acid (OA).

Supplement: Supplementary file 1 [file plants-13-02234-s001.zip › plants-3114673-supplementary.pdf]
